# Soluble Corin Predicts the Risk of Cardiovascular Disease: A 10-Year Follow-Up Study

**DOI:** 10.1016/j.jacasi.2022.01.004

**Published:** 2022-04-26

**Authors:** Linan Chen, Qiu Zhang, Min Zhang, Jia Yu, Liyun Ren, Jing Li, Shengqi Ma, Yan He, Weidong Hu, Hao Peng

**Affiliations:** a Department of Epidemiology, School of Public Health, Medical College of Soochow University, Suzhou, China; b Department of Chronic Disease, Gusu Center for Disease Control and Prevention, Suzhou, China; c Central Office, Suzhou National New and Hi-tech Industrial Development Zone Center for Disease Control and Prevention, Suzhou, China; d Department of Neurology, the Second Affiliated Hospital of Soochow University, Suzhou, China; e Jiangsu Key Laboratory of Preventive and Translational Medicine for Geriatric Diseases, Suzhou, China

**Keywords:** cardiovascular disease, corin, prospective longitudinal study, ANP, atrial natriuretic peptide, BNP, B-type natriuretic peptide, CHD, coronary heart disease, CVD, cardiovascular disease

## Abstract

**Background:**

As a key enzyme of the natriuretic peptides system, corin may participate in the development of cardiovascular disease (CVD). Its level in circulation predicted CVD recurrence in patients with myocardial infarction and heart failure, but no study examined this prediction in general populations.

**Objectives:**

This study sought to examine the prospective association between corin and CVD in a community-based population of Chinese adults.

**Methods:**

The Gusu cohort included 2,498 participants (mean age 53 years, 39% men) who were free of CVD at baseline. Serum corin was measured by enzyme-linked immunosorbent assay kits at baseline and CVD events were followed every 2 years for all participants. A competing-risks survival regression model was used to examine the association between serum corin and CVD.

**Results:**

During 10 years of follow-up, 210 participants developed CVD including 88 stroke events. A higher serum corin (after log-transformation) at baseline was significantly associated with an increased risk of CVD (HR: 1.88; *P =* 0.019) and stroke (HR: 3.19; *P =* 0.014). Analysis using categorical serum corin (in quartiles) showed that participants in the highest quartile had a 62% and 179% increased risk for CVD (HR: 1.62; *P =* 0.024) and stroke (HR: 2.79; *P =* 0.004), respectively, compared with those in the lowest quartile. We did not find a significant association between serum corin and coronary heart disease.

**Conclusions:**

A higher serum corin at baseline predicted a higher risk of CVD events and stroke, but not coronary heart disease, in Chinese adults, independent of conventional risk factors. Serum corin may be a predictor for stroke but the underlying mechanism needs further investigation.

Human corin, a type II transmembrane serine protease highly expressed in cardiac myocytes,^1^ is the physiological activator of atrial natriuretic peptide (ANP) and can also activate B-type natriuretic peptide (BNP), both of which are the main constitutes of the natriuretic peptides system that maintains blood pressure homeostasis through natriuresis, diuresis, and vasodilatation.^2^ As illustrated in the Central Illustration, corin may play a switching role in the natriuretic peptides system,^3^ thereby delivering an important impact on the homeostasis of the cardiovascular system. For example, animal studies found that transgenic mice with overexpression of corin had reduced myocardial fibrosis,^4^ and mice with the *corin* gene knockout developed cardiac hypertrophy and heart failure.^5^ In humans, single-nucleotide variations in the *CORIN* gene that encodes corin protease have been associated with hypertension.^6^, ^7^, ^8^ In published reports, corin protein was found to be shed from the cardiac myocyte surface by hydrolysis and autocleavage physiologically.^9^ Shed corin molecules could apparently enter the circulation and were found to possess the same activity in activating ANP as the membrane-bound corin was.^10^. Although the correlation between levels of corin in the circulation and expression on membrane-anchored corin is unclear, some small clinical studies have found that soluble corin was associated with cardiovascular disorders, such as atrial fibrillation,^11^ heart failure,^12^ and myocardial infarction.^13^ Our previous community-based case-control study also found a significant association between serum soluble corin and stroke.^14^ However, these findings are cross-sectional, and the temporal sequence between corin and cardiovascular disease (CVD), which is of considerable importance for causal inferences, is unknown. To date, 3 prospective studies have examined whether soluble corin at baseline could predict the future risk of CVD in patients with heart failure,^15^ acute myocardial infarction,^16^ and coronary heart disease (CHD),^17^ but the results are mixed. Moreover, circulating corin has been already changed in patients with cardiovascular disorders,^18^ the prospective association between soluble corin and CVD in such specific patients may therefore differ from that in general populations. However, this prospective association has not been studied in general populations free of overt CVD at baseline. Therefore, we aimed to examine whether soluble corin at baseline could predict the risk of CVD events after 10 years of follow-up in 2,498 community members who are free of CVD at baseline examination in the Gusu cohort. The associations of serum corin with specific CVD incidence, such as stroke and CHD, were additionally examined.

**Central Illustration:**
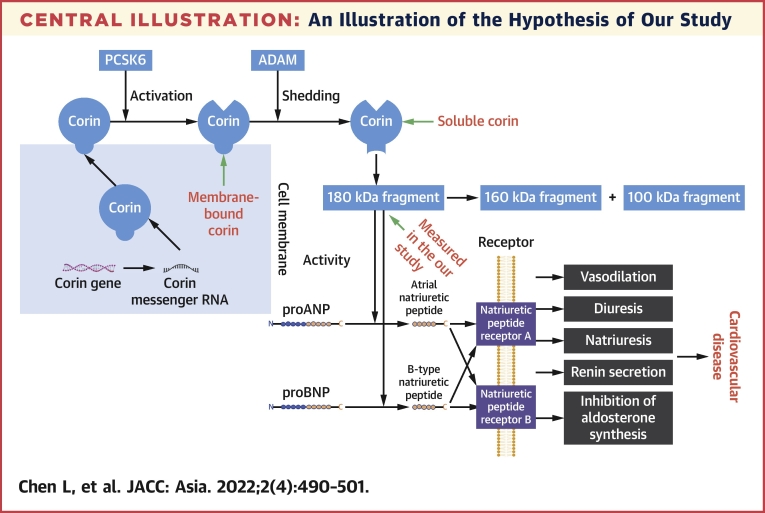
An Illustration of the Hypothesis of Our Study Corin can activate atrial natriuretic peptide (ANP) and B-type natriuretic peptide (BNP), both of which are the main constitutes of the natriuretic peptides system that maintains blood pressure homeostasis through natriuresis, diuresis, and vasodilatation. It may play a switching role in the natriuretic peptides system, thereby delivering an impact on the cardiovascular system. Corin protein was found to be shed from the cardiac myocyte surface. Shed corin molecules could apparently enter the circulation and were found to possess the same activity in activating ANP as the membrane-bound corin. Therefore, we speculated that serum corin may be associated with cardiovascular disease (CVD). mRNA = messenger RNA; NPRA = natriuretic peptide receptor A; NPRB = natriuretic peptide receptor B.

## Methods

### Study participants

The Gusu cohort is a community-based prospective longitudinal study of CVD and its risk factors in middle-aged and elderly Chinese adults. The study design, survey methods, and laboratory techniques have been described previously^19^ and in the Supplemental Appendix. In brief, a total of 2,498 community members over the age of 30 years and free of overt CVD were recruited and completed the baseline examination conducted in 2010. Hereafter, all participants were followed up every 2 years for new CVD events through 2020. The protocols were approved by the Soochow University Ethics Committee. Written informed consent was obtained from all study participants.

### Measurement of soluble corin at baseline

Blood samples were obtained by venipuncture in the morning after a requested overnight fast (at least 8 hours). All serum samples were separated and frozen at −80 °C until laboratory testing. Serum soluble corin measurements were performed in the Jiangsu Key Laboratory of Preventive and Translational Medicine for Geriatric Diseases by staff who were blind to the clinical characteristics of the study participants. All the samples were processed in a duplicate assay using a Quantikine human corin immunoassay (catalog: DCRN00, R&D Systems, Inc). A standard curve was constructed and from which corin concentrations of unknown samples were determined. Intra- and interassay coefficients of variation were <2.7% and <6.3%, respectively. Soluble corin was reported to be stable in blood samples frozen at −80 °C after several cycles of freezing and thawing.^20^

### Assessment of risk factors at baseline

Demographic data (age, sex, and education level), lifestyles (cigarette smoking, alcohol consumption), and metabolic factors (obesity, blood pressure, glucose, and lipids) were obtained at baseline. The detailed methods of data collection were presented in the Supplemental Appendix.

### Follow-up and assessment of cardiovascular events

CVD events in our study included nonfatal CHD (including acute myocardial infarction and unstable angina), nonfatal stroke, and death from any CVD causes. All of the participants were followed up by either phone calls or face-to-face visits by staff from the local community health service institutes every 2 years. The staff reviewed the hospital records and completed a standard event form when a new CHD, stroke, or death was identified during follow-up. Based on the event form, an endpoint review committee made the final diagnosis. The date of each event was ascertained from either the initial point of diagnosis or a death certificate. If an individual developed CHD and stroke successively, the first occurred event was counted as the CVD event, whereas both events were used in the analysis of specific CVD events, such as stroke and CHD.

### Statistical analysis

Baseline characteristics of study participants were presented according to quantiles of serum corin. Due to the sex difference in serum corin, study participants were categorized according to the distribution of serum corin in men and women individually. Log-transformation was applied to maximal normality of serum corin and the generated values (log-corin) were used in downstream analyses. To examine whether serum corin at baseline predicted the incidence of CVD events during follow-up, we constructed a competing-risks survival regression model in which time (in years) to incident CVD was the dependent variable, serum corin at baseline (log-corin as a continuous variable or quartiles as a categorical variable) was the independent variable, and death from causes other than CVD was the competing event, adjusting for age, sex, education level, current smoking, current drinking, body mass index, systolic blood pressure, low-density lipoprotein cholesterol, high-density lipoprotein cholesterol, fasting glucose, and antihypertensive medications (yes/no) at baseline. For participants who were lost or remained free of CVD by the end of follow-up, the time to events was censored at the end of follow-up. The rationale of using the competing-risks survival regression model was to account for the influence of death on the occurrence of CVD events,^21^ because participants may die for other causes before suffering any CVD events. The cumulative incidence of CVD events in the 4 groups of serum corin levels was estimated using the cumulative incidence functions and compared using the Fine-Gray test. To ease data interpretation, partial effect plots with spline curves were captured to visualize the impact of serum corin level on CVD events by constructing a restricted cubic spline regression model with 3 knots corresponding to the 35th, 65th, and 95th percentiles of serum corin. The associations of baseline serum corin with the incidence of stroke and CHD were similarly examined. The competing-risks survival regression models were constructed by the R package “cmprsk.”

### Sensitivity analysis

To examine whether sex affects our results, a sex-specific association between serum corin and CVD events was additionally examined. To examine whether serum corin improves prediction performance over conventional risk factors, such as age, sex, education level, current smoking, current drinking, body mass index, systolic blood pressure, low-density lipoprotein cholesterol, high-density lipoprotein cholesterol, fasting glucose, and antihypertensive medications, we established 2 Cox regression models (conventional risk factors only vs conventional risk factors plus log-corin), and then calculated and compared the area under the receiver-operating characteristic curves for 2 prediction models, which were implemented by the R package “timeROC.” All statistical analyses were conducted using R (version 4.0.3, R Foundation). A 2-tailed *P* value of <0.05 was considered statistically significant.

## Results

### Baseline characteristics

A total of 2,498 participants (mean age 53 years, 39% men) who were free of CVD at baseline in the Gusu cohort were included in the present study. Their baseline characteristics are shown in Table 1. Participants with a higher serum corin were more likely to be older and had more metabolic risk factors, such as obesity, blood pressure, fasting glucose, and lipids, than did those with a lower level of serum corin (all *P <* 0.05).

**Table 1:** Baseline Characteristics of Study Participants According to Serum Corin Levels (N = 2,498)

	Serum Soluble Corin				*P* Value for Trend
	Quartile 1 (n = 625)	Quartile 2 (n = 623)	Quartile 3 (n = 627)	Quartile 4 (n = 623)	
Age, y	52.4 ± 9.3	52.0 ± 9.6	53.2 ± 9.6	53.2 ± 9.7	0.035
Male	240 (38.40)	241 (38.68)	242 (38.60)	239 (38.36)	0.982
Education, high school or beyond	113 (18.08)	123 (19.74)	126 (20.10)	145 (23.27)	0.031
Current smoking	174 (27.84)	166 (26.44)	137 (21.85)	105 (16.85)	<0.001
Current drinking	129 (20.64)	126 (20.22)	113 (18.02)	97 (15.57)	0.012
Antihypertensive medications	116 (18.56)	132 (21.19)	165 (26.32)	210 (33.71)	<0.001
SBP, mm Hg	128.2 ± 17.2	127.8 ± 16.4	131.3 ± 17.2	133.0 ± 17.2	<0.001
DBP, mm Hg	83.6 ± 9.4	83.6 ± 8.8	85.3 ± 9.5	86.9 ± 8.9	<0.001
Body mass index, kg/m^2^	24.16 ± 3.65	24.26 ± 3.31	24.99 ± 3.52	25.72 ± 3.81	<0.001
LDL cholesterol, mmol/L	2.83 ± 0.77	2.95 ± 0.72	3.10 ± 0.76	3.11 ± 0.77	<0.001
HDL cholesterol, mmol/L	1.56 ± 0.56	1.52 ± 0.45	1.51 ± 0.36	1.44 ± 0.36	<0.001
Fasting glucose, mmol/L	5.24 ± 1.18	5.26 ± 1.07	5.46 ± 1.43	5.62 ± 1.57	<0.001
Total cholesterol, mmol/L	5.06 ± 2.03	5.17 ± 1.79	5.33 ± 2.00	5.30 ± 0.94	0.005
Triglycerides, mmol/L	1.35 ± 1.73	1.35 ± 1.3	1.43 ± 1.31	1.71 ± 1.89	<0.001

Values are mean ± SD or n (%) unless otherwise noted.

Quartile 1: ≤1,785.87 pg/mL for male subjects and ≤1,279.99 pg/mL for female subjects; quartile 2: 1,785.88∼2,174.46 pg/mL for male subjects and 1,280.00∼1,515.11 pg/mL for female subjects; quartile 3: 2,174.47∼2,646.72 pg/mL for male subjects and 1,515.12∼1,758.51 pg/mL for female subjects; quartile 4: ≥2,646.73 pg/mL for male subjects and ≥1,758.52 pg/mL for female subjects.

DBP = diastolic blood pressure; HDL = high-density lipoprotein; LDL = low-density lipoprotein; SBP = systolic blood pressure.

### Prospective association between serum corin and CVD events

**Figure 1:**
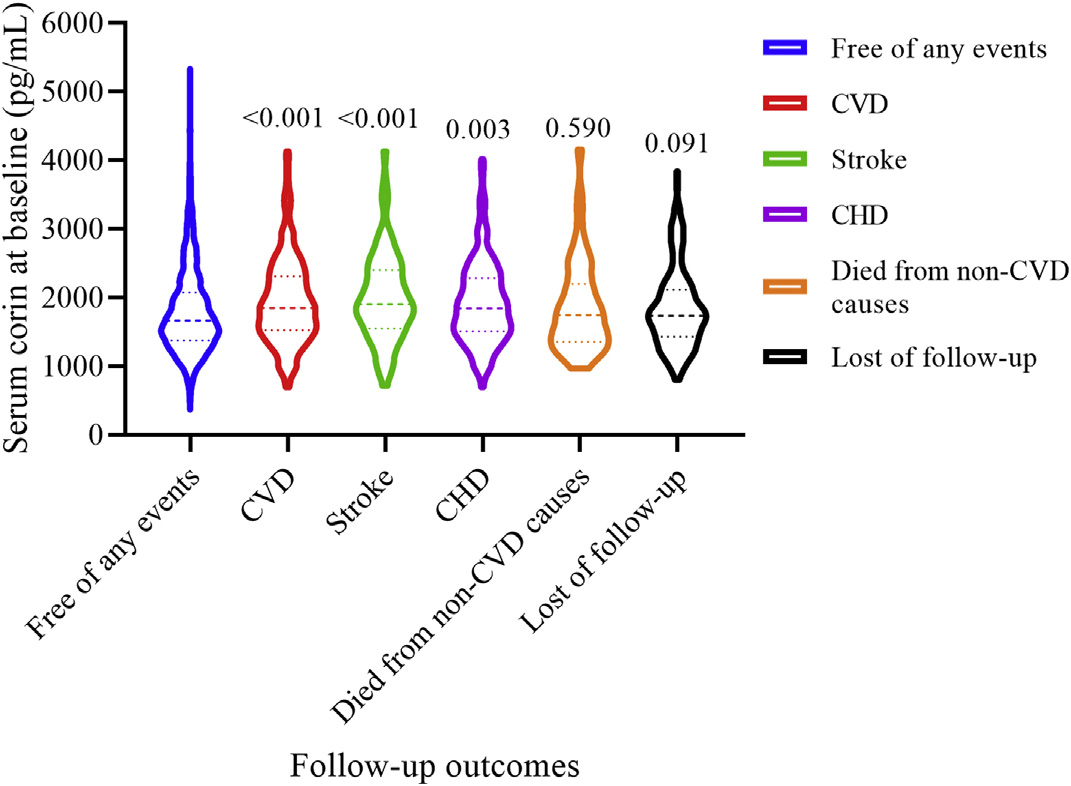
Serum Corin Levels for Participants With Different Outcomes During Follow-Up Compared with participants who remained free of any events (median: 1,665.2 pg/mL; IQR: 1,378.6-2,079.4 pg/mL), those who died of noncardiovascular causes (median: 1,750.1 pg/mL; IQR: 1,377.5-2,178.5 pg/mL; *P =* 0.590) or were lost to follow-up (median: 1,736.1 pg/mL; IQR: 1,441.6-2,111.7 pg/mL; *P =* 0.091) had a similar level of serum corin, whereas those who developed cardiovascular disease (CVD) (median: 1,850.5 pg/mL; IQR: 1,533.7-2,303.9 pg/mL; *P* < 0.001), stroke (median: 1,909.9 pg/mL; IQR: 1,557.9-2,400.5 pg/mL; *P* < 0.001), and coronary heart disease (CHD) (median: 1,846.3 pg/mL; IQR: 1,511.7-2,288.4 pg/mL; *P =* 0.003) had a significantly increased level of serum corin at baseline.

During an average of 10 years of follow-up, 210 participants developed new CVD events, 50 participants died of noncardiovascular causes, and 214 participants were lost (follow-up rate of 91.43%) (Table 2). Their baseline median levels of serum corin are shown in Figure 1. Compared with participants who remained free of CVD (median: 1,665.2 pg/mL; IQR:1,378.6-2,079.4 pg/mL), those who died of noncardiovascular causes (median: 1,750.1 pg/mL; IQR: 1,377.5-2,178.5 pg/mL) or were lost to follow-up (median: 1,736.1 pg/mL; IQR: 1,441.6-2,111.7 pg/mL) had a similar level of serum corin (all *P >* 0.05), whereas those who developed CVD (median: 1,850.5 pg/mL; IQR: 1,533.7-2,303.9 pg/mL) had a significantly increased level of serum corin at baseline (*P <* 0.001).

**Table 2:** Summary of the Follow-Up Outcomes of Study Participants in the Gusu Cohort (N = 2,498)

CVD events^a^	210
Nonfatal stroke^a^	81
Death from stroke	7
Nonfatal CHD^a^	139
Death from CHD	8
Death from non-CVD causes	50
Lost to follow-up	214
Survivors free of CVD	2,024

HD = coronary heart disease; CVD = cardiovascular disease.

a A total of 25 participants developed CHD and stroke successively during follow-up.

**Figure 2:**
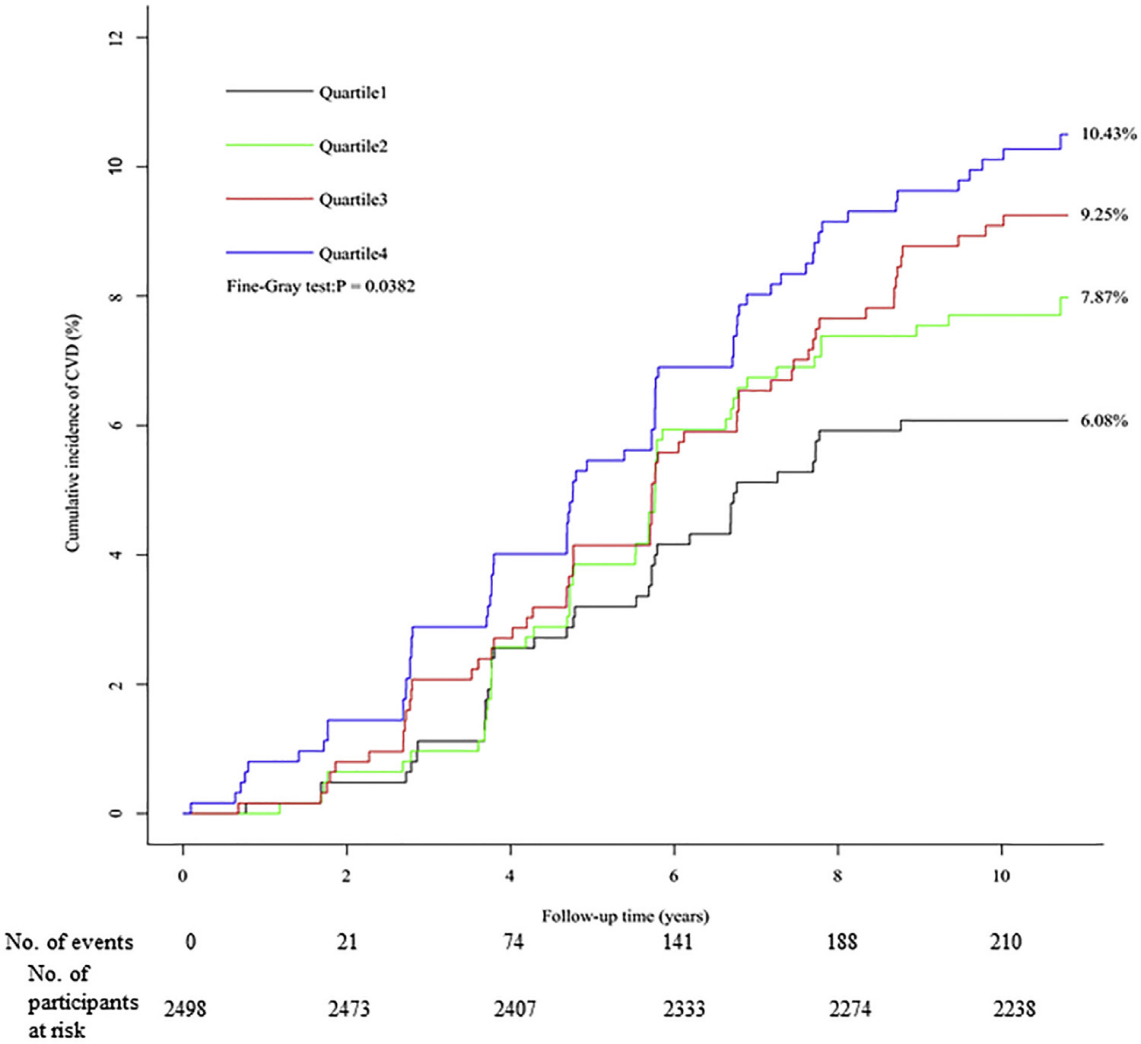
Cumulative Incidence of CVD According to Quartiles of Serum Corin The cumulative incidence of cardiovascular disease (CVD) was 6.08%, 7.87%, 9.25%, and 10.43% in participants with increasing quartiles of serum corin at baseline, respectively, with a significant group difference (*P =* 0.038 for the Fine-Gray test).

**Figure 3:**
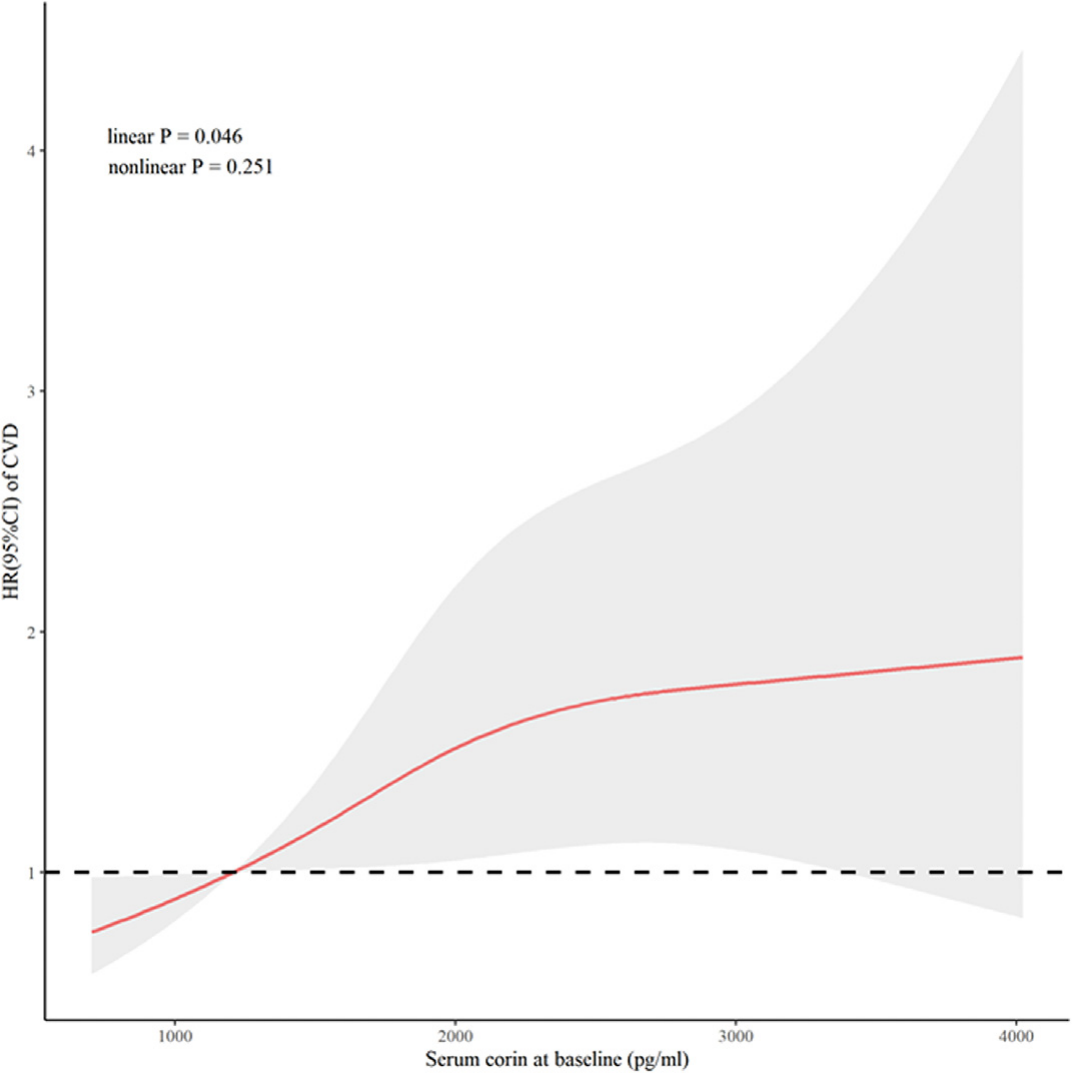
Cubic Spline Curves Visualizing the Impact of Corin on CVD HRs **(red line)** and their 95% CIs **(gray shading)** of cardiovascular disease (CVD) associated with baseline serum corin levels were calculated by constructing a restricted cubic spline regression model, after adjusting for age, sex, education level, current smoking, current drinking, systolic blood pressure, body mass index, low-density lipoprotein cholesterol, high-density lipoprotein cholesterol, fasting glucose, and antihypertension medications at baseline.

The cumulative incidence of CVD was 6.08%, 7.87%, 9.25%, and 10.43% in participants with increasing quartiles of serum corin at baseline, respectively, with a significant group difference (*P =* 0.038 for the Fine-Gray test) (Figure 2). After further adjustment for conventional risk factors, the association between serum corin at baseline and CVD was also observed. As shown in Table 3, the regression using log-corin as the independent variable revealed that a higher level of serum corin was significantly associated with an increased risk of CVD (HR: 1.88; *P =* 0.019). The regression using quartiles of serum corin as the independent variable found a similar association. Participants with upper quartiles of serum corin had 1.36 (*P =* 0.160), 1.39 (*P =* 0.130), and 1.62 (*P =* 0.024) times the risk of CVD as those with the lowest level of serum corin at baseline, although the first 2 HRs did not reach a statistically significant level. To ease data interpretation, the impact of serum corin level at baseline on the risk of CVD is also visualized in Figure 3.

**Table 3:** Prospective Associations of Baseline Serum Corin With Outcomes During Follow-Up

Serum Corin (pg/mL)	Events Unadjusted		Adjusted^a^		
	(n)	HR (95% CI)	*P* Value	HR (95% CI)	*P* Value
All CVD events					
Log-corin	210	2.12 (1.40-3.21)	<0.001	1.88 (1.11-3.18)	0.019
Categorical					
Quartile 1	38	1.00 (reference)		1.00 (reference)	
Quartile 2	49	1.30 (0.85-1.99)	0.220	1.36 (0.88-2.09)	0.160
Quartile 3	58	1.54 (1.02-2.31)	0.039	1.39 (0.91-2.13)	0.130
Quartile 4	65	1.75 (1.17-2.61)	0.006	1.62 (1.07-2.46)	0.024
*P* value for trend			0.004		0.029
Stroke					
Log-corin	88	2.58 (1.36-4.89)	0.004	3.19 (1.26-8.04)	0.014
Categorical					
Quartile 1	12	1.00 (reference)		1.00 (reference)	
Quartile 2	18	1.51 (0.73-3.13)	0.270	1.55 (0.74-3.27)	0.250
Quartile 3	22	1.84 (0.91-3.71)	0.091	1.60 (0.77-3.33)	0.210
Quartile 4	36	3.05 (1.59-5.86)	<0.001	2.79 (1.40-5.56)	0.004
*P* value for trend			<0.001		0.002
CHD					
Log-corin	147	1.86 (1.16-3.00)	0.011	1.51 (0.85-2.70)	0.159
Categorical					
Quartile 1	29	1.00 (reference)		1.00 (reference)	
Quartile 2	37	1.29 (0.79-2.09)	0.306	1.32 (0.81-2.16)	0.271
Quartile 3	42	1.45 (0.91-2.33)	0.121	1.33 (0.81-2.16)	0.259
Quartile 4	39	1.37 (0.85-2.21)	0.203	1.27 (0.77-2.09)	0.357
*P* value for trend			0.166		0.391
All-cause death					
Log-corin	65	2.04(0.96-4.31)	0.063	1.02(0.43-2.46)	0.989
Categorical					
Quartile 1	17	1.00 (reference)		1.00 (reference)	
Quartile 2	19	1.13 (0.59-2.18)	0.713	1.30 (0.67-2.52)	0.441
Quartile 3	17	1.01 (0.51-1.97)	0.985	1.03 (0.52-2.05)	0.934
Quartile 4	12	0.72 (0.34-1.51)	0.388	0.79 (0.37-1.70)	0.549
*P* value for trend			0.376		0.481

Quartile 1: ≤1,785.87 pg/mL for male subjects and ≤1,279.99 pg/mL for female subjects; quartile 2: 1,785.88∼2,174.46 pg/mL for male subjects and 1,280.00∼1,515.11 pg/mL for female subjects; quartile 3: 2,174.47∼2,646.72 pg/mL for male subjects and 1,515.12∼1,758.51 pg/mL for female subjects; quartile 4: ≥2,646.73 pg/mL for male subjects and ≥1,758.52 pg/mL for female subjects. CVD events included nonfatal CHD (including acute myocardial infarction and unstable angina), nonfatal stroke, and death from any CVD causes.

CHD = coronary heart disease; CVD = cardiovascular disease.

a Adjusted for age, sex, education level, current smoking, current drinking, systolic blood pressure, body mass index, low-density lipoprotein cholesterol, high-density lipoprotein cholesterol, fasting glucose, and antihypertension medications.

### Prospective association between serum corin and incident stroke

**Figure 4:**
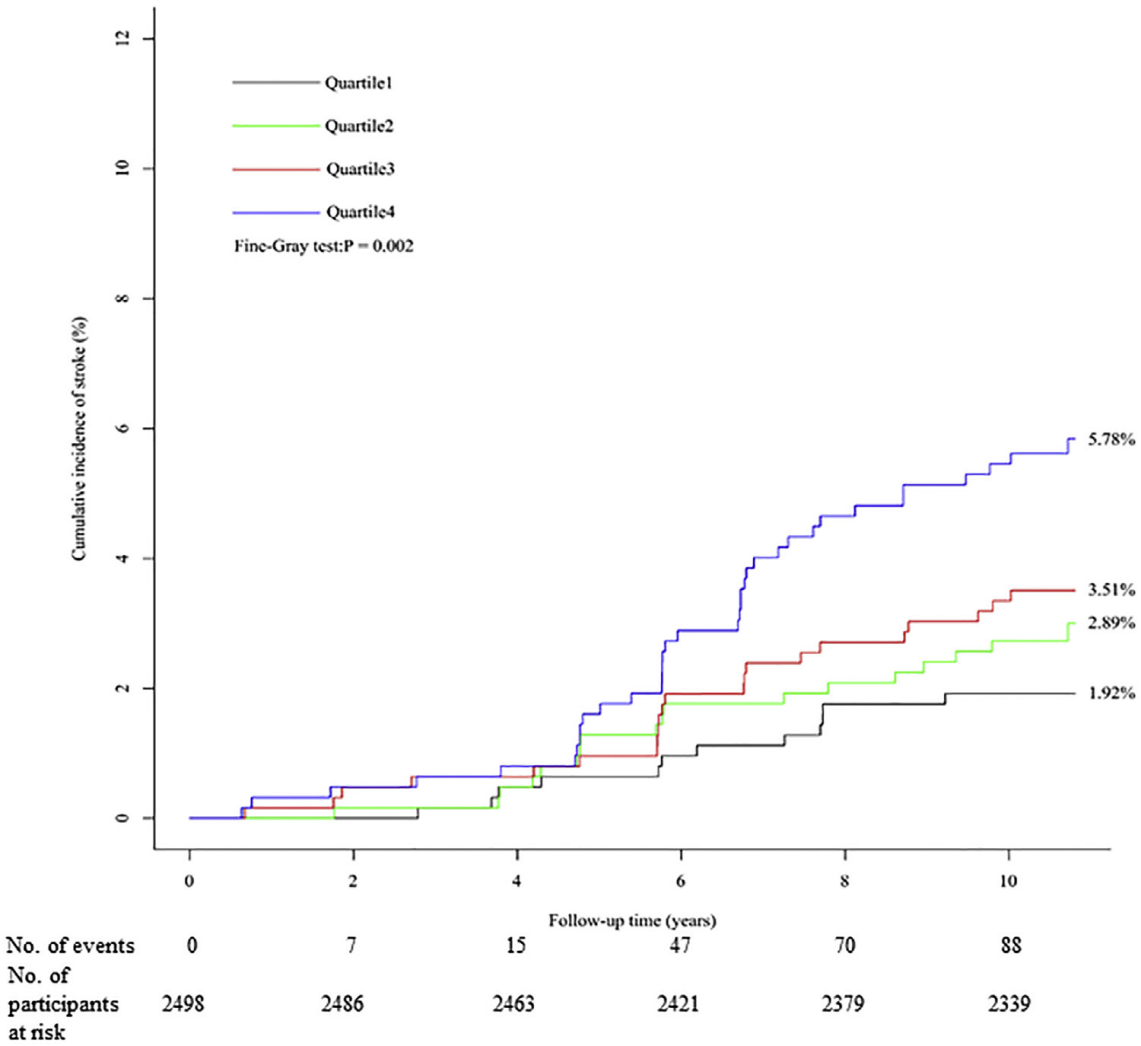
Cumulative Incidence of Stroke According to Quartiles of Serum Corin The cumulative incidence of stroke was 1.92%, 2.89%, 3.51%, and 5.78% in participants with increasing quartiles of serum corin at baseline, respectively, with a significant group difference (*P =* 0.002 for the Fine-Gray test).

**Figure 5:**
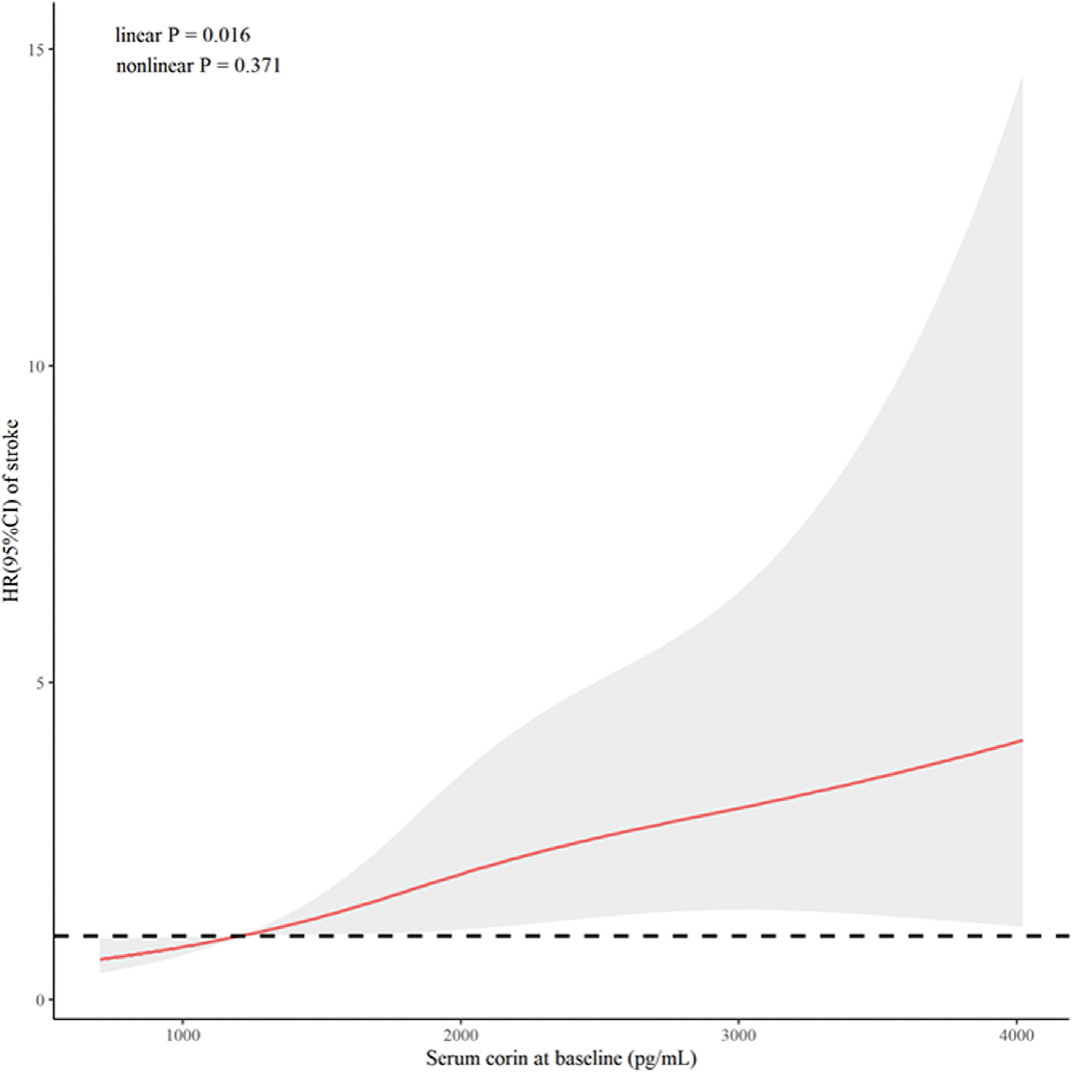
Cubic Spline Curves Visualizing the Impact of Corin on Stroke HRs **(red line)** and their 95% CIs **(gray shading)** of stroke associated with baseline serum corin levels were calculated by constructing a restricted cubic spline regression model, after adjusting for age, sex, education level, current smoking, current drinking, systolic blood pressure, body mass index, low-density lipoprotein cholesterol, high-density lipoprotein cholesterol, fasting glucose, and antihypertension medications at baseline.

A total of 88 stroke events were recorded during follow-up. Compared with those free of any events, participants who developed stroke during follow-up had a significantly increased level of serum corin at baseline (median: 1,909.9 pg/mL; IQR: 1,557.9-2,400.5 pg/mL; *P* < 0.001) (Figure 1). The cumulative incidence of stroke was 1.92%, 2.89%, 3.51%, and 5.78% in participants with increasing quartiles of serum corin at baseline, respectively, with a significant group difference (*P =* 0.002 for the Fine-Gray test) (Figure 4). Similarly, a higher level of serum corin at baseline was also significantly associated with a higher risk of stroke (HR: 3.19; *P =* 0.014 for log-corin), independent of conventional risk factors. Compared with participants with the lowest level of serum corin, those with the highest quartile of serum corin had a 179% (HR: 2.79; *P =* 0.004) higher risk of incident stroke (Table 2). As for specific subtype of stroke, the association between serum corin and ischemic stroke persisted (Supplemental Table 1). The impact of serum corin level at baseline on the risk of stroke is also visualized in Figure 5.

### Prospective association between serum corin and incident CHD

A total of 147 CHD events were identified during follow-up. Compared with those free of any events, participants who developed CHD during follow-up had a significantly increased level of serum corin at baseline (median: 1,846.3 pg/mL; IQR: 1,511.7-2,288.4 pg/mL; *P =* 0.003) (Figure 1). The cumulative incidence of CHD in participants with increasing quartiles of serum corin was 4.64%, 5.94%, 6.70%, and 6.26%, respectively, but without statistically significant difference (*P =* 0.451 for the Fine-Gray test) (Supplemental Figure 1). Univariate analysis found that serum corin at baseline was significantly associated with a higher risk of CHD (HR: 1.86; *P =* 0.011), but this association did not survive after adjusting for conventional risk factors (Table 3).

### Results of sensitivity analysis

Subgroup analysis by sex found that the associations between serum corin and CVD events persisted in men rather than in women but without significant heterogeneity (Supplemental Table 2). Adding serum corin to the prediction model did not significantly increase prediction performance for CVD events (AUC: 0.746 vs 0.744; *P =* 0.625), stroke (AUC: 0.796 vs 0.792; *P =* 0.652), or CHD (AUC: 0.729 vs 0.728; *P =* 0.707) compared with the model including conventional risk factors only (Supplemental Figure 2). We did not find significant association between serum corin and all-cause death (Table 3).

## Discussion

In the community-based prospective longitudinal study of middle-aged and elderly Chinese adults, we examined for the first time the prospective association between soluble corin at baseline and the future risk of CVD in a general population. We found that a higher level of serum corin at baseline predicted a higher risk of incident CVD during a 10-year follow-up. This association was independent of conventional risk factors, including behavioral and metabolic factors. As for specific CVD events, serum corin at baseline was also significantly associated with an increased risk of stroke, rather than CHD, during follow-up. The level of circulating corin could be a predictor of the risk of stroke. These findings suggest that corin may play a considerable effect on the cardiovascular system and may therefore serve as a candidate risk factor or a potential therapeutic target for stroke. Nevertheless, the causal effect of corin on stroke development still needs more evidence from trials.

In the present study, the serum corin levels of each participant were measured using commercial enzyme-linked immunosorbent assays, which are also widely used in other studies.^12^^,^^22^, ^23^, ^24^, ^25^ As an activator of the natriuretic peptides system, corin plays a critical role in maintaining blood pressure hemostasis through natriuresis, diuresis, and vasodilatation.^2^ Therefore, elevated blood pressure may need more corin to reduce the levels to normal. In our study, there were 1,109 hypertensive participants (44%) and serum corin was significantly higher in participants with hypertension than in those without.^19^ However, the range of serum corin levels (373.39-2,833.35 pg/mL) detected in nonhypertensive individuals in our study was similar to that (256-2,590 pg/mL) in previous studies.^20^

In line with our study, the identified association between serum corin and CVD has also been suggested by other studies. For example, a basic study reported an up-regulated expression of corin in human endothelial cells with atherosclerosis.^26^ In animals, the expression of the *corin* gene was up-regulated in hypertrophic cardiomyocytes and failing myocardium in mice.^27^ Plasma corin level was significantly increased in mice with myocardial infarction^28^ and heart failure,^29^ compared with in their wild littermates. In humans, some single-nucleotide variations of the coding gene of corin, such as rs111253292, rs3749585, and rs2271037, have been associated with the susceptibility of hypertension, which is the leading contributor of CVD.^6^^,^^8^^,^^30^^,^^31^ Furthermore, corin levels in circulation have also been associated with some cardiovascular disorders. For instance, a small clinical study including 141 patients with atrial fibrillation and 127 matched control subjects demonstrated that plasma corin was significantly increased in patients with atrial fibrillation.^11^ Another study found a significant association between plasma corin and infarct size in 55 patients with myocardial infarction.^32^ A recent prospective cohort study including 1,009 patients with heart failure found that a higher level of soluble corin and neprilysin at baseline was significantly associated with a higher risk of cardiovascular death and rehospitalization during 8 years of follow-up.^22^ Also, a high level of serum PCSK6, an upstream activator of corin, was found to be associated with an increased risk of CVD during a median follow-up of 2 years in 565 patients who had undergone coronary angiography.^17^ In addition, circulating corin was also significantly increased in the pregnant woman with hypertension and preeclampsia.^33^, ^34^, ^35^ In the Gusu cohort, our group has examined and found that serum corin was significantly and positively associated with major risk factors of CVD, such as hypertension,^19^ diabetes,^36^^,^^37^) dyslipidemia,^38^ and obesity.^39^ In the current study, we further examined whether serum corin at baseline could predict the future risk of CVD in the Gusu cohort. To the best of our knowledge, this is the first prospective cohort study to examine the association between corin and CVD in a general population. Our findings enrich the published data on the potential role of corin in the cardiovascular system. Together with prior studies, the significant prospective association observed in our study increases the possibility that corin might be a candidate risk factor and a therapeutic target for cardiovascular disorders.

In contrast, a negative association between corin and CVD has also been observed in prior studies. For example, transgenic mice with overexpression of corin had a lower risk of myocardial fibrosis and heart failure.^4^^,^^40^ Mice with the *corin* gene knockout developed hypertension and cardiac hypertrophy.^5^ Case-control studies found that circulating corin was lower in patients with stroke,^14^ myocardial infarction,^13^ and heart failure^12^^,^^41^ than in their matched healthy control subjects. Prospective cohort studies found that a lower level of plasma corin at admission was associated with a higher risk of CVD during a follow-up of 5 years in patients with chronic heart failure^15^ and acute myocardial infarction.^16^ Another prospective study including 565 patients undergoing coronary angiography did not find a significant association between corin and CVD.^17^ The reasons for the inconsistent findings are unclear. One of the possible explanations could be the varied populations studied: most of the prospective cohort studies included patients who had already suffered from CVD, whereas our study included community members who were free of CVD at baseline. As an activator of ANP and BNP, corin acts as an upstream regulator of the natriuretic peptides system. This system plays a critical role in maintaining salt-water balance and blood pressure through natriuresis, diuresis, and vasodilation in response to cardiac output overload. As a result, corin expression and excretion may be up-regulated in compensatory myocytes for high-risk individuals of CVD, such as hypertensive patients, because these individuals may need more ANP and BNP to reduce their blood pressure. Our previous studies have demonstrated that serum corin was elevated in participants with CVD risk factors such as hypertension,^19^ diabetes,^36^^,^^37^ dyslipidemia,^38^ obesity,^39^ and metabolic syndrome.^42^ In such conditions, a higher level of corin at baseline may indicate a higher risk of CVD in the future. On the other hand, the expression of corin may not increase in the patients who were decompensated and who already had a myocardial infarction and heart failure. The activity of corin on activation of ANP has been reduced in patients with heart failure.^18^^,^^29^ Therefore, a negative association between corin and the recurrence of CVD may be observed in a cohort of patients with myocardial infarction and heart failure, as discussed.^15^^,^^16^ Indeed, mountains of evidence have demonstrated that higher natriuretic peptides predicted a higher risk of cardiovascular events.^43^, ^44^, ^45^ Higher levels of natriuretic peptides require more corin protease to activate them because corin is the physical activator of ANP. These findings further support our results that a higher level of serum corin predicts a higher risk of future incidence of CVD for participants who are free of CVD at baseline.

In addition to population studies, basic experiments are also encouraged to deepen our understanding of the role of corin in the cardiovascular system. Corin has been found to play critical roles in the natriuretic peptides system by activating ANP and BNP,^3^ energy metabolism by degrading agouti-related protein,^46^ and suppression of oxidative stress.^47^ These known mechanisms suggest that the function of corin is complicated and it may participate in the development of CVD through multiple pathways. Whether corin possesses any more biological functions that may affect the cardiovascular system is still yet to be answered. A better understanding of the underlying molecular mechanisms would undoubtedly promote the clinical translation of corin shortly.

To the best of our knowledge, our study represents the first to examine the prospective association between baseline serum corin and CVD incidence in a general population who were free of CVD at baseline. The strengths of our study include the prospective longitudinal study design with 10 years of follow-up, the comprehensive adjustment of conventional risk factors, and the application of a competing-risks survival regression model to account for the bias introduced by deaths from noncardiovascular causes.

### Study limitations

First, as an observational study, we cannot eliminate residual confounding. The causality between serum corin and CVD is not established and needs further evidence from clinical trials. However, our prospective design conformed the temporality that elevated corin preceded the incidence of CVD and thereby increasing the possibility of the causality. Second, our study mainly included middle-aged and elderly Chinese adults, the generalization of our results to younger and other ethnic populations would be cautious. Third, we did not have data on natriuretic peptides in our study and cannot determine whether and how serum corin was associated with natriuretic peptides. However, 2 studies reported that serum corin was associated with ANP and BNP levels assessed by their equimolarly cleaved forms N-terminal proANP^48^ and N-terminal proBNP,^49^ respectively. These results increased the possibility that corin may play a role in cardiovascular health. Fourth, small clinical studies have found that soluble corin was associated with atrial fibrillation^11^ and heart failure.^12^ These disease states may therefore affect the association between serum corin and CVD. Unfortunately, we did not collect data on atrial fibrillation and heart failure at baseline. Whether and how these disease states affect our results is unclear.

## Conclusions

Our study demonstrated that higher serum corin at baseline predicted a higher risk of CVD events and stroke, but not CHD, in an unselected community-based population of Chinese adults, independent of conventional risk factors.

Perspectives **COMPETENCY IN MEDICAL KNOWLEDGE:** Soluble corin has been found to predict the risk of cardiovascular events in patients with myocardial infarction and heart failure. However, this prospective association has not been examined in general populations. Our study for the first time found that a higher level of serum corin at baseline could predict a higher future risk of incident stroke in Chinese adults. Serum corin may be a useful biomarker to identify individuals at a high risk of stroke in primary prevention.

**TRANSLATIONAL OUTLOOK:** The causality between corin and stroke still needs more evidence and the underlying molecular mechanisms are also waiting to be studied.

## Funding Support and Author Disclosures

This study was supported by the National Natural Science Foundation of China (82173596, 81903384, and 81872690), the Natural Science Foundation of Jiangsu Province (BK20180841), the Suzhou Municipal Science and Technology Bureau (SKJY2021040 and SYS2020091), the Youth Program of Science and Technology for Invigorating Health through Science and Education in Suzhou (KJXW2020084), and a Project of the Priority Academic Program Development of Jiangsu Higher Education Institutions. The authors have reported that they have no relationships relevant to the contents of this paper to disclose.
