# Supplementary material for: Soluble Corin Predicts the Risk of Cardiovascular Disease: A 10-Year Follow-Up Study
Source: JACC Asia. 2022 Apr 26;2(4):490–501. doi: 10.1016/j.jacasi.2022.01.004 (PMC9627939; doi:10.1016/j.jacasi.2022.01.004)
Supplement: Supplemental Data [file mmc1.docx]

**Soluble Corin Predicts the Risk of Cardiovascular Disease: A 10-year follow-up study**

**Running title:** corin and cardiovascular events

Linan Chen ^1*^, MD; Qiu Zhang ^2*^, MD; Ming Zhang ^3^, MD, PhD; Jia Yu ^1^, MD; Liyun Ren ^1^, MD; Jing Li ^1^, MD; Shengqi Ma ^1^, MD; Yan He ^1^, MD, PhD; Weidong Hu ^4†^, MD, PhD; Hao Peng ^1,5†^, MD, PhD.

**Supplemental data**

**Study participants**

The Gusu cohort is a community-based prospective longitudinal study of CVD and its risk factors in middle-aged and elderly Chinese adults. The study design, survey methods, and laboratory techniques have been described previously (1). In brief, 8 communities were randomly selected as the research fields from the 39 communities in Gusu district in 2010. All eligible participants residing in these fields were invited to participate if they were aged over 30 years, with a Han ethnicity, and had lived in the area for at least 10 years. There were a total of 3,061 eligible residents in the study fields, but only 2,706 (participating rate: 88%) individuals agreed to participate in this study. After providing written informed consent, they received questionnaires and were offered free physical examination and clinical biochemical tests using blood and urine specimens under the principle of voluntary acceptance. Based on the information obtained, 208 participants were excluded from the cohort if they met at least one of the following criteria: (i) having clinical suspicion of diseases which may cause secondary hypertension (e.g., renal artery stenosis, coarctation, glomerulonephritis, pyelonephritis, pheochromocytoma, Cushing’s syndrome, Conn’s syndrome), (ii) self-reported history of CHD, stroke, or tumors, (iii) self-reported thyroid or parathyroid diseases, (iv) being pregnant, and (v) lacking blood samples. A total of 2,498 participants completed the baseline examination and were finally enrolled in the Gusu cohort study. Hereafter, all participants were followed up every two years for new CVD events through 2020. The protocols were approved by the Soochow University Ethics Committee.

**Data collection at baseline**

Demographic data including age, sex, and education level were obtained by questionnaires administered by trained staff. Cigarette smoking was defined as current smoking or not. Current smoking was defined as having smoked at least 100 cigarettes in the entire life, having smoked cigarettes regularly, and smoking currently. Alcohol consumption was classified as current drinkers or not. Current drinkers were those who had consumed any alcoholic beverage ≥ 12 times during the past year. Three blood pressure measurements were performed by trained staff using a standard mercury sphygmomanometer and a cuff of appropriate size, according to a common protocol adapted from procedures recommended by the American Heart Association (2), after the participants had been resting for at least 5 min in a relaxed, sitting position. The first and fifth Korotkoff sounds were recorded as systolic blood pressure (SBP) and diastolic blood pressure (DBP), respectively. The means of the three measurements were used for statistical analyses. Body weight (kg) and height (cm) were measured when participants wore light clothes and no shoes by trained staff. Body mass index (BMI) was calculated by dividing weight in kilograms by the square of height in meters (kg/m^2^). Fasting glucose, blood lipids including total cholesterol, triglycerides, high-density lipoprotein cholesterol (HDL-C), and low-density lipoprotein cholesterol (LDL-C), were measured by standard laboratory methods.

**Reference**:

1. Peng H, Zhang Q, Cai X et al. Association Between High Serum Soluble Corin and Hypertension: A Cross-Sectional Study in a General Population of China. American journal of hypertension 2015;28:1141-9.

2. Pickering TG, Hall JE, Appel LJ et al. Recommendations for blood pressure measurement in humans: an AHA scientific statement from the Council on High Blood Pressure Research Professional and Public Education Subcommittee. J Clin Hypertens (Greenwich) 2005;7:102-109.

| **Supplemental Table S1**. Prospective associations of baseline serum corin with the risks of ischemic and hemorrhagic stroke | | | | | |
| --- | --- | --- | --- | --- | --- |
| Serum corin (pg/mL) | No. of events | Unadjusted | | Adjusted* | |
|  |  | HR (95% CI) | *P* value | HR (95% CI) | *P* value |
| Ischemic stroke |  |  |  |  |  |
| Log-corin | 77 | 2.56(1.28-5.13) | 0.008 | 3.09(1.12-8.55) | 0.030 |
| Categorical |  |  |  |  |  |
| Quartile 1 | 9 | 1.00 (reference) |  | 1.00 (reference) |  |
| Quartile 2 | 16 | 1.79(0.79-4.05) | 0.161 | 1.83(0.55-4.20) | 0.157 |
| Quartile 3 | 20 | 2.23(1.02-4.89) | 0.046 | 1.84(0.80-4.27) | 0.153 |
| Quartile 4 | 32 | 3.62(1.73-7.57) | <0.001 | 3.19(1.45-7.01) | 0.004 |
| *P* for trend |  |  | <0.001 |  | 0.002 |
| Hemorrhagic stroke |  |  |  |  |  |
| Log-corin | 11 | 2.62(0.56-12.39) | 0.224 | 3.60(0.46-27.95) | 0.221 |
| Categorical |  |  |  |  |  |
| Quartile 1 | 3 | 1.00 (reference) |  | 1.00 (reference) |  |
| Quartile 2 | 2 | 0.67(0.11-3.99) | 0.657 | 0.70(0.12-4.02) | 0.688 |
| Quartile 3 | 2 | 0.66(0.11-3.97) | 0.652 | 0.76(0.15-3.88) | 0.736 |
| Quartile 4 | 4 | 1.34(0.30-5.97) | 0.705 | 1.55(0.34-7.01) | 0.571 |
| *P* for trend |  |  | 0.714 |  | 0.583 |
| ^*^Adjusted for age, education level, current smoking, current drinking, systolic blood pressure, body mass index, low-density lipoprotein cholesterol, high-density lipoprotein cholesterol, fasting glucose, and antihypertension medication. | | | | | |

| **Supplemental Table S2**. Prospective associations of baseline serum corin with incident CVD, stroke, and CHD in men and women | | | | | | | | |  |
| --- | --- | --- | --- | --- | --- | --- | --- | --- | --- |
| CVD event | | Unadjusted | *P*-value | *P* for heterogeneity |  | Adjusted^*^ | *P*-value | *P* for heterogeneity | |
|  |  | HR (95% CI) |  |  |  | HR (95% CI) |  |  |  |
| All CVD events | | | | | | | | | |
|  | Men | 1.89 (0.97-3.66) | 0.061 | 0.628 |  | 2.26(1.07-4.74) | 0.034 | 0.587 | |
|  | Women | 2.46 (1.11-5.43) | 0.026 |  |  | 1.37(0.60-3.16) | 0.454 |  |  |
| Stroke | | | | | | | | | |
|  | Men | 2.13 (0.61-7.36) | 0.234 | 0.250 |  | 2.94(0.71-12.21) | 0.140 | 0.818 | |
|  | Women | 6.02 (1.79-20.26) | 0.004 |  |  | 3.32(0.88-12.50) | 0.076 |  |  |
| CHD | | | | | | | | | |
|  | Men | 1.72 (0.87-3.39) | 0.120 | 0.973 |  | 2.04(0.96-4.32) | 0.064 | 0.483 | |
|  | Women | 1.68 (0.67-4.20) | 0.265 |  |  | 1.02(0.39-2.65) | 0.970 |  |  |
| ^*^Adjusted for age, education level, current smoking, current drinking, systolic blood pressure, body mass index, low-density lipoprotein cholesterol, high-density lipoprotein cholesterol, fasting glucose, and antihypertension medication.  CVD: cardiovascular disease; CHD: coronary heart disease; HR: hazards ratio; CI: confidence interval. | | | | | | | | | |


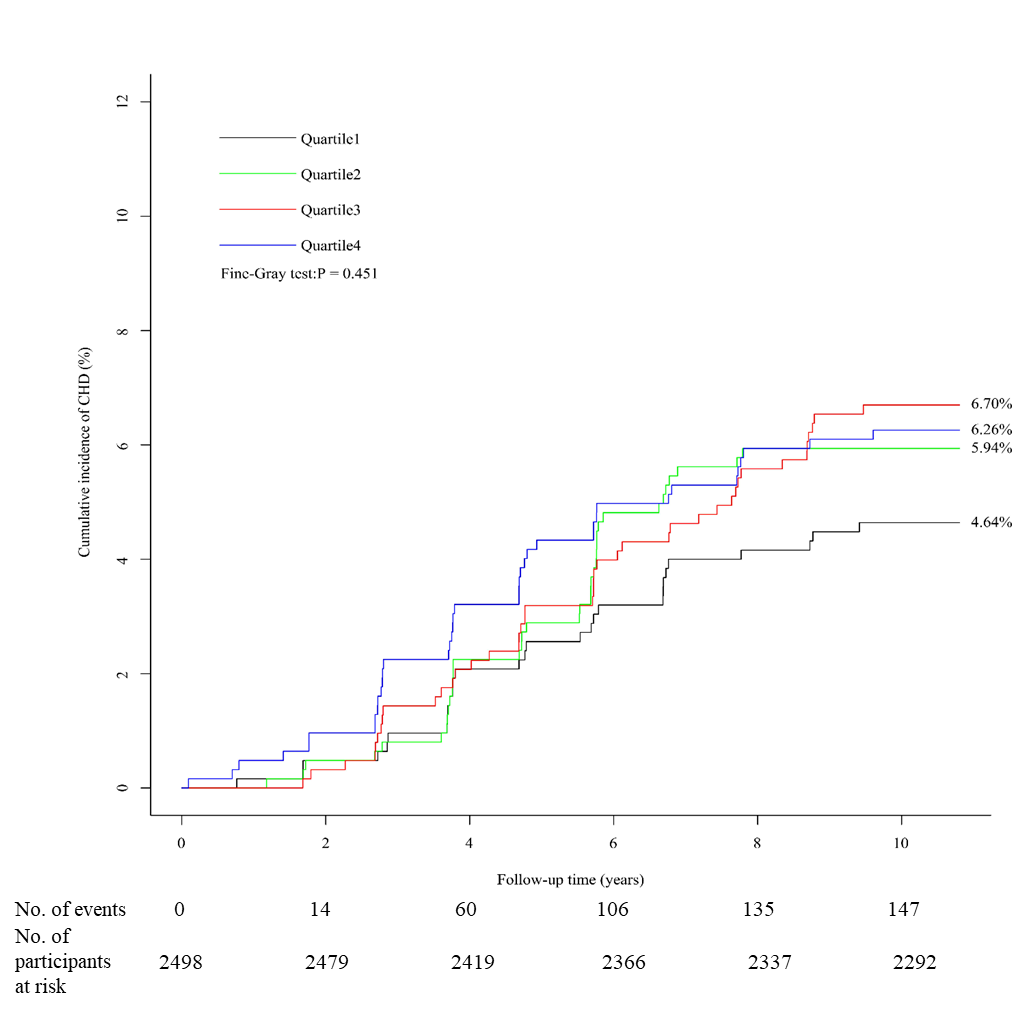
**Supplemental Figure S1.** The cumulative incidence of CHD for participants with different levels of serum corin (in quartiles) at baseline. The cumulative incidence of CHD in participants with increasing quartiles of serum corin was 4.64%, 5.94%, 6.70%, and 6.26%, respectively, but without statistically significant difference (*P*=0.451 for the Fine-Gray test). CHD: coronary heart disease.


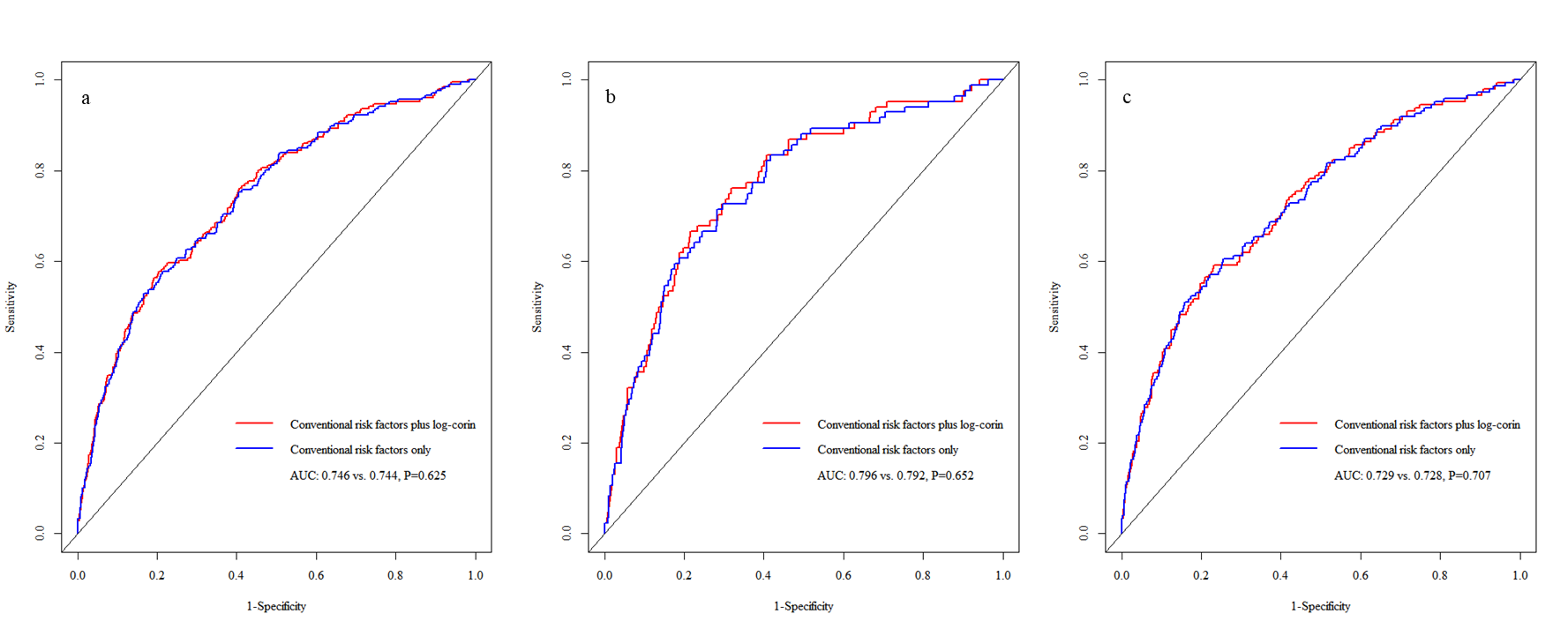
**Supplemental Figure S2.** ROC curves illustrating the prediction performance of conventional risk factors and serum corin for CVD (a), stroke (b), and CHD (c).
